# Stapler versus manual closure for pharyngeal repair after total laryngectomy: a systematic review and meta-analysis

**DOI:** 10.1007/s00405-026-10129-8

**Published:** 2026-03-09

**Authors:** Noof Albannai, Munawer Alsaeed, Yaqoub Yousef Alenezi, Bander M. Alshammari, Nawaf Almotairi, Noora Albuainain, Retaj Alawadhi, Abdullah M. Alharran, Mishal Almutairi

**Affiliations:** 1 School of Medicine, Trinity College Dublin, Dublin, Ireland; 2 College of Medicine and Medical Sciences, Arabian Gulf University, Manama, Kingdom of Bahrain; 3 Otorhinolaryngology-Head and Neck Surgery, Kuwait Institute for Medical Specializations (KIMS), Kuwait City, Kuwait; 4 Department of Otolaryngology–Head and Neck Surgery, Farwania Hospital, Kuwait City, Kuwait

**Keywords:** Total Laryngectomy, Pharyngocutaneous Fistula, Stapler Closure, Manual Suture, Pharyngeal Repair, Meta-Analysis

## Abstract

**Introduction:**

Pharyngeal closure is a critical step in total laryngectomy influencing postoperative recovery. While manual suturing is traditional, stapler-assisted closure offers potential advantages. This study provides an updated meta-analysis comparing stapler versus manual closure, stratifying by surgical indication, laryngectomy type and stapler technique.

**Methodology:**

Following PRISMA guidelines, a systematic search of four databases, up to November 2025, was conducted. Randomized controlled trials (RCTs) and observational studies comparing stapler versus manual closure in total laryngectomy were included. The primary outcome was pharyngocutaneous fistula (PCF) incidence. Secondary outcomes mainly included operative time and hospital stay. Risk ratio (RR) or mean difference (MD) with 95% confidence intervals (CI) were calculated using a random-effects model.

**Results:**

Twenty studies (4 RCTs, 16 observational) involving 1,545 patients were included. Stapler closure was associated with a significantly lower risk of PCF (RR = 0.54, 95% CI [0.38, 0.76], *p* < 0.001), shorter operative time (MD = -52.60, 95% CI [-74.33, -30.86], *p* < 0.001), and reduced hospital stay (MD = -3.62, 95% CI [-5.49, -1.76], *p* < 0.001). Subgroup analysis demonstrated a significant PCF reduction in primary laryngectomy (RR = 0.40, 95% CI [0.24, 0.69], *p* > 0.001), but this benefit was not statistically significant in salvage laryngectomy alone. Closed and semi-closed stapler techniques showed the most significant protective effects.

**Conclusion:**

Stapler-assisted pharyngeal closure significantly reduces PCF incidence, operative time, and hospitalization compared to manual suturing in primary laryngectomy. While efficient, its superiority in preventing PCF in high-risk salvage settings is not established, warranting careful patient selection.

**Supplementary Information:**

The online version contains supplementary material available at 10.1007/s00405-026-10129-8.

## Introduction

Total laryngectomy remains a cornerstone in the management of advanced or recurrent laryngeal cancer [1, 2]. A critical, time-consuming step of this procedure is the closure of the pharyngeal defect to create a functional neopharynx [3]. The integrity of this closure is essential, as failure can lead to pharyngocutaneous fistula (PCF), a complication associated with increased morbidity, prolonged hospitalization, and delayed adjuvant therapy [4, 5]. While traditional manual, hand-sewn suturing has long been the standard of care, mechanical linear staplers have emerged as a promising alternative, offering the potential for a faster, more standardized, and watertight closure [6].

Current evidence regarding the superiority of one technique over the other remains controversial [7]. Several systematic reviews, including those by Lee et al. [8] and Chiesa-Estomba et al. [9], have suggested that stapler closure is associated with shorter operative times, reduced hospital stays, and a lower incidence of PCF. However, these meta-analyses were limited by significant methodological heterogeneity, often pooling data from both randomized controlled trials (RCTs) and observational studies. Moreover, they lacked detailed subgroup analyses to investigate the influence of main clinical variables, such as the surgical setting (primary vs. salvage laryngectomy) or the specific stapler application technique (e.g., closed, semi-closed, or open). This lack of detailed analysis leaves a critical gap in the evidence, and prevents the development of context-specific clinical guidelines [10]. To address these limitations, our study presents an updated and more comprehensive systematic review and meta-analysis. By incorporating a larger patient cohort from the most recent literature and performing extensive subgroup analyses stratified by study design, laryngectomy type, and stapler technique, we aim to provide a more robust evaluation. Therefore, this study aims to clarify the comparative effectiveness of stapler versus manual pharyngeal closure in total laryngectomy, providing higher-quality evidence to guide surgical decision-making.

## Methodology

### Protocol and registration

This systematic review and meta-analysis were designed and executed in accordance with the Preferred Reporting Items for Systematic Reviews and Meta-Analyses (PRISMA) 2020 statement and Cochrane handbook [11, 12]. The study protocol was established a priori to define the research question, search strategy, inclusion criteria, and analytical plan.

(CRD420251240350).

## Eligibility criteria

We established eligibility criteria based on the Population, Intervention, Comparator, and Outcomes (PICO) framework to guide study inclusion. We included RCTs and observational cohort studies that enrolled adult patients undergoing total laryngectomy for any indication (e.g., primary or salvage treatment for laryngeal cancer). The intervention of interest was pharyngeal closure performed with a mechanical linear stapler. The comparator was conventional pharyngeal closure using a manual, hand-sewn suturing technique. Studies were required to report on at least one of our predefined outcomes to be eligible for inclusion. We excluded case reports, review articles, conference abstracts, and studies that did not provide a distinct comparator group or sufficient data for quantitative analysis.

### Information sources and search strategy

A systematic literature search was conducted across four electronic databases: PubMed, Cochrane Library, Scopus, and Web of Science, from their inception until November 2025, with no language restrictions. Our search strategy combined keywords and subject headings related to the core concepts of our research, including terms such as “total laryngectomy,” “pharyngeal closure,” “stapler,” “mechanical suture,” and “hand-sewn.” The full search syntax applied to each database is detailed in the Supplementary Table 1. To ensure a comprehensive retrieval of relevant literature, we also manually screened the reference lists of all included articles and pertinent review papers for any additional eligible studies.

### Study selection

The study selection was performed in a two-stage screening process. First, two independent reviewers screened all retrieved titles and abstracts to identify potentially relevant articles. Records deemed irrelevant by both reviewers were excluded. In the second stage, the full texts of the remaining articles were assessed for final eligibility against the predefined inclusion criteria. Any disagreements between the reviewers at either stage were resolved through discussion or, if necessary, by consulting a senior reviewer.

### Data extraction

A standardized data extraction template was developed to systematically capture the main information from each included study. Two reviewers independently extracted the following data: **(1) Study Characteristics**: First author, publication year, country, study design, time frame, total sample size, inclusion and exclusion criteria, laryngectomy type, stapler type, closure type, manual closure description, follow-up duration, and study conclusion. **(2) Population Details**: study ID, study groups, total sample size, mean age, sex distribution, diabetes mellitus, alcohol use, smoking history, prior irradiation, previous tracheotomy, neck dissection, and full T-stage distribution (T0–T4).

### Risk of bias assessment

The methodological quality and risk of bias of the included studies were independently evaluated by two reviewers using design-appropriate tools. For RCTs, we utilized the Revised Cochrane Risk-of-Bias 2 (RoB-2) tool [13]. For observational cohort studies, the Newcastle-Ottawa Scale (NOS) was employed, which assesses studies based on patient selection, comparability of groups, and outcome assessment [14]. Any discrepancies in the assessments were resolved by consensus.

### Outcome measures

The primary outcome of this meta-analysis was the incidence of postoperative PCF. Secondary outcomes included: (1) total operative time, (2) length of hospital stay, (3) time to initiate oral feeding, and (4) incidence of surgical wound infection.

### Statistical analysis

All statistical analyses were conducted using R statistical software (version 4.3.1) with the “meta” package. For dichotomous outcomes, we calculated Risk Ratios (RR) with 95% confidence intervals (CI). For continuous outcomes, Mean Differences (MD) with 95% CI were computed. Given the anticipated clinical and methodological diversity among the studies, we employed a random-effects model (DerSimonian and Laird method) for all pooled analyses to account for potential between-study heterogeneity.

Heterogeneity was quantified using the I² statistic, with values of < 25%, 25%–75%, and > 75% interpreted as low, moderate, and high heterogeneity, respectively. A p-value < 0.10 from the chi-squared test was considered indicative of significant heterogeneity. We explored sources of heterogeneity through prespecified subgroup analyses based on study design (RCT vs. cohort), laryngectomy type (primary, salvage, or mixed), and stapler application technique (open, closed, semi-closed, and “mainly closed technique and semi-closed technique if needed”).

To assess the stability of our findings, a leave-one-out sensitivity analysis was performed for each outcome. Publication bias was visually inspected using funnel plots and formally assessed using Egger’s regression test. A two-sided p-value of less than 0.05 was considered statistically significant for all analyses except for the test of heterogeneity.

## Results

### Literature search results

**Fig. 1:**
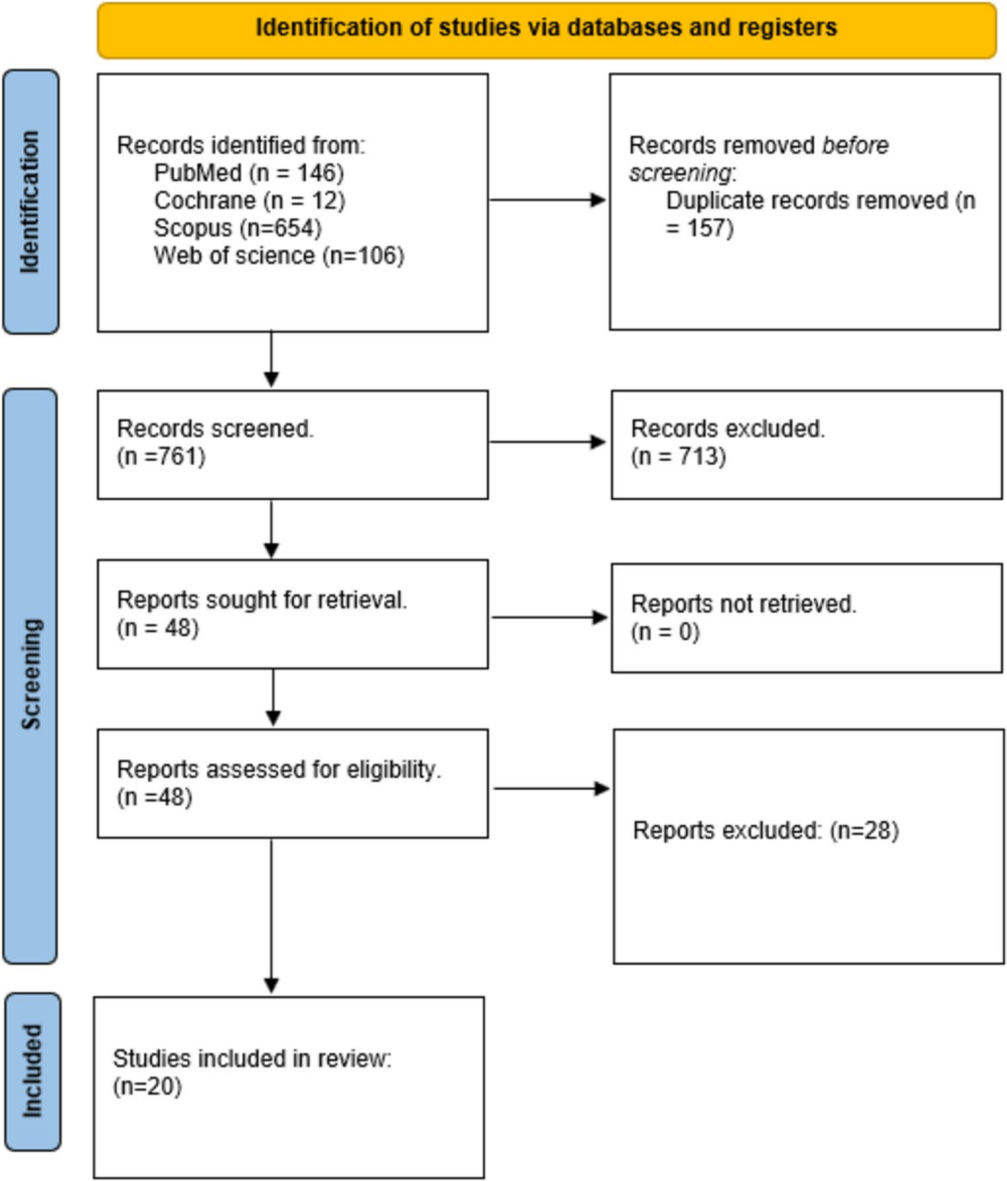
PRISMA flow diagram regarding the selection process of the included studies

Our initial search across four databases, PubMed, Cochrane, Scopus, and Web of Science, identified 918 records. After the removal of 157 duplicates, 761 unique records were screened based on their titles and abstracts, leading to the exclusion of 713 records. The full texts of the remaining 48 reports were retrieved and assessed for eligibility. Following a detailed review, 28 reports were excluded for not meeting the inclusion criteria. Finally, 20 studies were included in the qualitative and quantitative synthesis. The study selection process is detailed in the PRISMA flowchart (Fig. 1).

### Characteristics of Included Studies

The 20 included studies were published between 2009 and 2025 and consisted of four RCTs and 16 observational studies [3, 4, 6, 7, 10, 15–29]. Geographically, the studies were conducted in Italy, Egypt, Turkey, Brazil, the USA, Thailand, India, China, Jordan, Spain, and Poland. A total of 1,545 patients were analyzed, with 632 undergoing stapler closure and 913 manual closure. The patient population across the studies was predominantly male, with a mean age generally ranging from the late 50 s to the late 60s. The clinical context of the laryngectomies varied: 14 studies included a mixed cohort of both primary and salvage laryngectomies, four focused exclusively on primary laryngectomy, and two investigated salvage laryngectomy alone. The proportion of patients with prior irradiation also varied significantly across studies.

The intervention group received pharyngeal closure using linear staplers of various lengths (ranging from 45 mm to 90 mm), with the majority of studies employing a closed or semi-closed technique. The control group underwent manual, hand-sewn closure, for which the techniques were heterogeneous, including two- or three-layer repairs, T-shaped or vertical closures, and the use of various suture materials such as Vicryl, Dermalon, or gut.

Across the studies, the primary inclusion criterion was patients with laryngeal squamous cell carcinoma undergoing total laryngectomy. Common exclusion criteria were tumour extension into the hypopharynx, base of the tongue, or pyriform sinus, as well as the need for complex flap reconstruction (e.g., local or free flaps). Detailed characteristics of the included studies and their populations are summarized in Tables 1 and 2.

**Table 1:** Summary characteristics of the included studies

Study ID	Study design	Country	Time frame	Total Sample Size		Inclusion criteria	Exclusion criteria	Laryngectomy type	Stapler type	Closure type	Manual closure description	Follow up, months	Conclusion
** Parrilla 2025**	Retrospective cohort study	Italy	2012 to 2022	110	Patients with a histological diagnosis of squamous cell carcinoma of the larynx who underwent TL and VP placement.		Patients with involvement of the posterior paraglottic space on CT, pharyngeal involvement, different tumor histotypes, distant metastases, non-resectable tumors, secondary laryngeal neoplasms, and complex flap reconstruction.	Patients with primary and salvage laryngectomy	Vertical closure with 60 mm linear stapler and horizontal closure with 45 mm linear stapler (T-shaped repair)	Closed technique (Hybrid Primary Puncture)	Traditional open total laryngectomy with hand-sewn pharyngeal closure in two layers (mucosa + pharyngeal constrictor muscle).	-	The Hybrid Primary Puncture technique in stapler-assisted total laryngectomy enables simultaneous stapler closure and primary voice prosthesis insertion without increasing operative risk.
** Galazka 2025**	Retrospective cohort study	Poland	January 2020 to January 2025	52	Patients who underwent total laryngectomy for laryngeal cancer.		Patients exhibiting significant tissue fibrosis or scarring, particularly those who had undergone high-dose pre-RTH, were generally assigned to the manual group, implying exclusion from the stapler group based on tissue integrity.	Patients with primary and salvage laryngectomy	2 rows 45 or 60 mm linear stapler	Open technique	Two-layer hand-sewn closure with non-absorbable Dermalon 3.0: first layer interrupted, second running; tongue base sutured to esophageal mucosa, then lateral walls approximated.	0.69	Although manual suturing remains the standard closure method after total laryngectomy, stapler-assisted closure demonstrates lower pharyngocutaneous fistula rates and represents a reliable alternative for esophageal reconstruction.
** Nosiglia 2025**	Retrospective cohort study	Italy	November 2013 to December 2023	70	Patients with advanced squamous cell carcinoma of the larynx (according to TNM staging) who underwent TL.		Patients who had received previous radiotherapy, chemotherapy, or open neck surgery (except tracheotomy for respiratory failure). Patients with Tumors involving the base of tongue, vallecula, postcricoid area, pre-epiglottic area, pyriform fossa, or hypopharynx were also excluded.	Patients with primary laryngectomy only	60 mm linear stapler	Semi-closed technique	Traditional 3-layered manual suture (T-type)	0.33	The findings indicate that mechanical stapler closure following total laryngectomy may provide distinct benefits in selected patients, including lower pharyngocutaneous fistula rates, shorter hospitalization, and reduced operative duration. Beyond statistical significance, these outcomes carry notable clinical relevance, potentially enhancing postoperative recovery and optimizing the use of surgical resources.
** Teaima 2025**	Retrospective cohort study	Egypt	2015 to 2022	91	Patients with laryngeal squamous cell carcinoma who underwent total laryngectomy. Patients with advanced laryngeal carcinoma (T3 or T4a) in cases of primary laryngectomy or any stage in salvage laryngectomy.		Patients with extralaryngeal extension, those needing flap reconstruction (local or free flap), and patients with multiple comorbidities.	Patients with primary and salvage laryngectomy	75- or 80-mm linear stapler	Closed technique	Vertical 3-layer hand-sewn pharyngeal repair using Vicryl 4/0 and 3/0 on a round needle.	24	Stapler use in total laryngectomy provides a simple, rapid, and efficient closure technique, offering tension-free, watertight sealing, superior hemostasis, and reduced contamination of the surgical field compared with manual suturing.
** Paulose 2024**	Retrospective cohort study	India	July 2017 to July 2023	71	Patients with diagnosed advanced endolaryngeal squamous cell carcinoma without pharyngeal mucosal extension who underwent total laryngectomy in a primary or salvage setting.		Patients with advanced endolaryngeal squamous cell carcinoma exhibiting pharyngeal mucosal extension, or with tumor spread into the hypopharynx or pre-epiglottic space.	Patients with primary and salvage laryngectomy	60 mm linear stapler	Closed technique	Traditional repair of the neopharynx using continuous Connell suture with 3 − 0 absorbable polyglactin or 3 − 0 braided polyester.	22.28	Stapler-assisted closure can be effectively applied in endolaryngeal tumor cases, providing reduced operative duration and improved surgical efficiency, while achieving oncologic outcomes equivalent to those of conventional hand-sewn closure techniques.
** Mandor 2024**	RCT	Egypt	February 2021 to February 2022	60	Patients with advanced laryngeal carcinoma (T3 and T4)		Patients with tumor extension to the hypopharynx or the tongue base, or those with positive surgical margins.	Patients with primary and salvage laryngectomy	2 row 60 mm linear stapler	Closed technique in 24/30 cases; semi-closed in 6/30	Two-layer hand-sewn closure: mucosa closed with running inverting 3/0 Vicryl, then constrictor muscles sutured as a second layer; cricopharyngeal (constrictor) myotomy done to improve swallowing.	24	The stapler represents a reliable technique for pharyngeal closure following total laryngectomy, provided that its indications relative to the primary tumor are appropriately respected. Its use is associated with a lower incidence of pharyngocutaneous fistula, shorter operative time, and favorable swallowing outcomes, achieved without compromising oncological safety.
** Han 2024**	Retrospective cohort study	China	January 2018 to December 2021	66	Patients diagnosed with laryngeal squamous cell carcinoma requiring total laryngectomy, who underwent pharyngeal closure using manual suturing, a linear stapling device, or a thyroid gland flap, and who presented with cervical lymph node metastasis.		Patients who had received radiotherapy, chemotherapy, or targeted therapy within one month before surgery, or who had distant metastasis, additional primary malignancies, poorly controlled systemic diseases, or received complex flap reconstructions (PMMC or free flap).	Patients with primary and salvage laryngectomy	75 mm linear stapler	Closed technique	Y-shaped, two-layer mucosal closure: mucosa 3 − 0 Vicryl interrupted (15–20 stitches), then soft tissue/muscle layers with 2 − 0 Vicryl, plus subcutaneous and skin layers.	60	The thyroid gland flap is a safe and effective option for repairing mucosal defects and closing the pharyngeal cavity following total laryngectomy. However, in cases without mucosal defects or extensive tumor invasion, linear stapler closure remains the most time-efficient and practical technique.
** Salzano 2023**	Retrospective cohort study	Italy	January 2006 to January 2021	91	Patients with histologically confirmed endolaryngeal squamous cell carcinoma (ELSCC) who experienced primary treatment failure (after radiotherapy, chemoradiotherapy, or prior surgery) and had no involvement of the tongue base, hypopharynx, or extralaryngeal structures.		Patients were excluded from the stapler group if they had posterior tumor extension, infiltration of the suprahyoid epiglottis, pyriform sinus, or retrocricoid region, or if the case was not endolaryngeal SCC.	Patients with salvage laryngectomy only	Linear stapler	Semi-closed technique	Conventional hand-sewn pharyngeal closure	0.82	Stapler-assisted pharyngeal closure after total laryngectomy is recommended for endolaryngeal tumors, as it appears to reduce pharyngocutaneous fistula incidence, operative time, hospital stay, and time to oral feeding. However, larger studies are needed to validate these findings and establish a standardized surgical protocol.
** Algargaz 2022**	Retrospective cohort study	Jordan	January 2014 to December 2018	59	Patients with tumors well-contained within the boundaries of the larynx and those with anterior extra-laryngeal extension underwent total laryngectomy.		Patients with tumors involving the base of the tongue, vallecula, pyriform sinus, or who had a preoperative tracheostomy.	Patients with primary and salvage laryngectomy	3 row 60 mm linear stapler	Closed and semi-closed techniques were used.	The pharyngeal defect was closed with interrupted 3/0 vicryl sutures.	-	Both suturing techniques showed no statistically significant difference in pharyngocutaneous fistula (PCF) rates; however, operative time was notably shorter with stapler use compared to manual suturing. Further large-scale randomized studies are required to confirm these findings and establish the definitive role of stapler closure in pharyngeal repair.
** Ahmed 2022**	RCT	Egypt	April 2017 to March 2018	60	Patients with tumors confined to the endolarynx who are undergoing total laryngectomy.		Patients with tumor extension to the hypopharynx, base of the tongue, or pyriform fossa.	Patients with primary and salvage laryngectomy	60 mm linear stapler	Closed technique	Three-layer hand-sewn closure: (1) mucosa closed with 3 − 0 Vicryl running Connell suture, (2) submucosa + muscle closed with 3 − 0 Vicryl running suture, (3) cricopharyngeus edges approximated (standard open, inverting manual closure).	12	Stapler-assisted pharyngeal closure is technically straightforward and equally effective as traditional hand-sewn neopharyngeal suturing in patients undergoing total laryngectomy.
** Galli 2021**	Retrospective cohort study	Italy	January 2004 to June 2018	114	Patients affected by endolaryngeal squamous cell carcinoma who underwent salvage total laryngectomy following primary treatment failure.		Patients with extralaryngeal extension, involvement of the tongue base, or hypopharyngeal tumors were excluded. Exclusion criteria for stapler use included posterior paraglottic space infiltration and arytenoid cartilage sclerosis.	Patients with salvage laryngectomy only	Vertical closure with 60 mm linear stapler and horizontal closure with 45 mm linear stapler (T-shaped repair)	Closed technique and semi-closed if needed	Conventional hand-sewn pharyngeal closure	0.39	The use of a stapler was associated with reduced operative time and hospital stay, as well as an earlier resumption of oral feeding. Additionally, mechanical pharyngeal closure appears to lower the incidence of pharyngocutaneous fistula, although its preventive effect in salvage laryngectomy requires confirmation through further research.
** Sansa-Perna 2020**	Retrospective cohort study	Spain	2008 to 2018	126	Patients with tumors confined to the endolarynx who are undergoing total laryngectomy.		Patients were excluded from the mechanical suturing technique if the tumor involved the aryepiglottic folds, suprahyoid epiglottis, inter-arytenoid/retro-cricoid areas, or medial wall of pyriform sinuses, due to oncological risk.	Patients with primary and salvage laryngectomy	75 mm linear stapler	Closed technique	Hypopharyngeal mucosa sutured with 4 − 0 Vicryl; usually a horizontal suture to the base of the tongue; in irradiated, a T-shaped suture is used to reduce tension; a second layer routinely added, then the constrictor muscle placed over as reinforcement.	60	Mechanical pharyngeal suturing following total laryngectomy is an oncologically safe and reliable technique, provided it is applied in appropriately selected cases.
** Öztürk 2019**	RCT	Turkey	August 2014 to June 2016	41	Patients had biopsy-proven laryngeal cancer who needed total laryngectomy.		Patients were excluded from the mechanical suturing technique if the tumor involved the aryepiglottic folds, suprahyoid epiglottis, inter-arytenoid/retro-cricoid areas, or the medial wall of the pyriform sinus.	Patients with primary laryngectomy only	60 mm linear stapler	Closed technique	Open pharyngeal closure with interrupted 3/0 Vicryl sutures	-	The linear stapler technique in total laryngectomy is a safe, reliable, and time-efficient method that is easy to perform. It significantly reduces pharyngeal closure time without adversely affecting nasogastric tube removal or pharyngocutaneous fistula rates.
** Galletti 2018**	RCT	Italy	March 2003 to February 2014	43	Patients with advanced laryngeal carcinoma (T3-T4, N0-3, M0-1) undergoing total laryngectomy.		Patients with tumor size and local invasion that did not allow adequate pharyngeal suturing.	Patients with primary and salvage laryngectomy	90 mm linear stapler	Closed technique	Conventional manual suture with needle and gut	-	Compared with conventional manual closure, the use of mechanical suturing significantly reduces operative time and hospital stay, facilitates earlier restoration of oral feeding and removal of the nasogastric tube, and is associated with a lower incidence of postoperative complications, including fistula formation and fever.
** Ismi 2017**	Prospective cohort study	Turkey	January 2014 to May 2016	70	Patients who underwent TL operation due to laryngeal squamous cell carcinoma.		Patients with hypopharyngeal tumors and tumors reconstructed with complex flaps (PMMC or free flap). Also, patients requiring esophageal mucosa excision or closure with tension were excluded from the manual closure group.	Patients with primary and salvage laryngectomy	2 row 80 mm linear stapler	Semi-closed technique	T-type manual esophageal closure with 3/0 Vicryl	-	Stapler-assisted esophageal closure reduces operative time, pharyngocutaneous fistula (PCF) incidence, surgical site infection (SSI) rates, and NNISS scores, but does not influence systemic complication rates. Comorbidities and prolonged surgery are key risk factors for systemic complications; however, the presence of comorbid illness alone should not guide stapler use solely to minimize postoperative systemic risks.
** Dedivitis 2014**	Prospective cohort study	Brazil	1996 to 2011	87	Patients with laryngeal squamous cell carcinoma who underwent total laryngectomy.		Patients with hypopharyngeal carcinoma, extralaryngeal tumors, or tumors involving the base of the tongue were excluded from the stapler group. Patients in the control group with tension in the suture line not eligible for primary closure were also excluded.	Patients with primary and salvage laryngectomy	2 row 75 mm linear stapler	Closed technique and semi-closed if needed	Conventional manual pharyngeal suture	-	The use of a stapler does not increase the incidence of pharyngocutaneous fistula.
** Miles 2013**	Retrospective cohort study	The USA	January 2002 to December 2007	42	Patients with a history of advanced-stage cancer whose surgical defects were suitable for primary closure and who subsequently underwent total laryngectomy.		Patients were excluded if they required locoregional or free-tissue transfer reconstruction of the neopharynx.	Patients with primary and salvage laryngectomy	60–90 mm linear stapler	Closed technique	Hand-sewn Connell technique	-	In appropriately selected patients, primary neopharyngeal closure can be safely and effectively accomplished using a linear stapling device, ensuring both technical efficiency and oncological safety.
**Sannikorn 2013**	Retrospective cohort study	Thailand	January 2007 to December 2011	52	Patients with a diagnosis of laryngeal cancer, deemed suitable for surgical management, and showing no involvement of adjacent structures, including the pyriform fossa, aryepiglottic fold, post-cricoid region, epiglottis, or base of the tongue.		Patients requiring flap reconstruction, with recurrent or secondary cancers, a previous history of head and neck cancer surgery, or tumor involvement of adjacent structures such as the pyriform fossa or base of the tongue.	Patients with primary laryngectomy only	2 row 60 mm linear stapler	Closed technique	Two-layer hand-sewn: (1) mucosa/fascia running suture with inversion; (2) muscle layer interrupted.	-	Pharyngeal stapling may serve as a viable alternative technique for pharyngeal closure in total laryngectomy procedures.
** Calli 2011**	Prospective cohort study	Turkey	May 2002 to April 2009	182	Patients underwent total laryngectomy.		Patients with hypopharyngeal carcinoma, extralaryngeal tumor extension, or tumor involvement of the base of the tongue, as well as those who had received any form of preoperative radiotherapy.	Patients with primary laryngectomy only	2 row 60 mm linear stapler	Closed technique	Conventional manual pharyngeal suture	0.23	Mechanical stapler closure of the pharynx following total laryngectomy was associated with a significant reduction in pharyngocutaneous fistula incidence compared with manual suturing, particularly in appropriately selected cases.
** Gonçalves 2009**	Prospective cohort study	Brazil	March 2001 to May 2005	60	Patients with endolaryngeal squamous cell carcinoma who underwent total laryngectomy.		-	Patients with primary and salvage laryngectomy	2 row 75 mm linear stapler	Closed technique	Linear transverse hand-sewn pharyngeal closure	-	Mechanical closure in total laryngectomy is a simple, quick, and reliable technique that ensures watertight closure and minimal contamination, remaining safe even in irradiated patients when applied within appropriate tumor extension limits confirmed intraoperatively.

Total laryngectomy (TL), Voice prosthesis (VP), Randomized controlled trial (RCT), Pharyngocutaneous fistula (PCF), Radiotherapy (RTH), Chemoradiotherapy (CRT), Squamous cell carcinoma (SCC), Endolaryngeal squamous cell carcinoma (ELSCC), Partial myocutaneous pectoralis major flap (PMMC), Nasogastric tube (NGT), Neck Infection Severity Score (NNISS), Hypopharyngeal cancer (HPC), Linear stapling device (LSD)

**Table 2:** Baseline characteristics of the included studies

Study ID	Groups	N	Age (y), mean (SD)	Sex (male), N (%)	Diabetes Mellitus, N (%)	Alcohol consumption, N (%)	Smoking, N (%)	Prior irradiation, N (%)	Previous tracheotomy, N (%)	Neck dissection, N (%)	T-stage				
											T0	T1	T2	T3	T4
** Parrilla 2025**	Stapler closure	43	65.1 (6.5)	93 (84.55)	-	-	-	-	-	43 (100)	-	-	-	-	-
	Manual closure	67			-	-	-	-	-	67 (100)	-	-	-	-	-
** Galazka 2025**	Stapler closure	29	59.8 (9.6)	44 (84.6)	-	-	-	13 (25)	-	-	0 (0)	1 (2)	3 (5.8)	20 (38.4)	28 (53.8)
	Manual closure	23													
** Nosiglia 2025**	Stapler closure	36	77.76 (8.4)	31 (86.1)	-	-	-	0 (0)	7 (19)	-	0 (0)	0 (0)	5 (13.9)	21 (58.3)	10 (27.8)
	Manual closure	34	72.97 (9.34)	30 (88.2)	-	-	-	0 (0)	10 (29)	-	0 (0)	0 (0)	3 (8.8)	15 (44.1)	16 (47.1)
** Teaima 2025**	Stapler closure	54	58.73 (7.84)	50 (92.59)	-	-	-	-	16 (29.63)	-	-	-	-	-	-
	Manual closure	37	59.41 (6.64)	35 (94.59)	-	-	-	-	11 (29.73)	-	-	-	-	-	-
** Paulose 2024**	Stapler closure	18	58 (7)	18 (100)	-	4 (22)	10 (56)	4 (22)	-	-	0 (0)	0 (0)	2 (11)	8 (44)	8 (44)
	Manual closure	53	57 (9)	52 (98)	-	13 (25)	40 (75)	24 (45)	-	-	1 (2)	0 (0)	6 (12)	21 (40)	24 (46)
** Mandor 2024**	Stapler closure	30	60.53 (9.43)	-	9 (30)	-	-	7 (23.3)	5 (16.7)	30 (100)	0 (0)	0 (0)	0 (0)	5 (16.7)	25 (83.3)
	Manual closure	28	58.61 (5.74)	-	10 (35.7)	-	-	6 (21.4)	8 (28.6)	28 (100)	0 (0)	0 (0)	0 (0)	8 (28.6)	20 (71.4)
** Han 2024**	Stapler closure	16	63.5 (7.44)	-	-	-	-	-	-	-	-	-	-	-	-
	Manual closure	50	62.96 (9.29)	-	-	-	-	-	-	-	-	-	-	-	-
** Salzano 2023**	Stapler closure	42	66.81	37 (88.1)	7 (16.67)	7 (16.67)	42 (100)	23 (54.76)	-	39 (92.86)	0 (0)	0 (0)	3 (7.14)	8 (19.05)	31 (73.81)
	Manual closure	49	65.73	45 (91.8)	8 (16.33)	5 (10.2)	47 (95.9)	24 (48.98)	-	45 (91.84)	0 (0)	0 (0)	2 (4.08)	12 (24.49)	35 (71.43)
** Algargaz 2022**	Stapler closure	22	60.5	98 (98.3)	-	-	-	-	0 (0)	-	-	-	-	-	-
	Manual closure	37	59.9		-	-	-	-	0 (0)	-	-	-	-	-	-
** Ahmed 2022**	Stapler closure	30	56.2 (7.09)	28 (93.3)	18 (60)	-	-	-	-	30 (100)	0 (0)	0 (0)	0 (0)	22 (73.3)	8 (26.7)
	Manual closure	30	55.9 (7.82)	26 (86.7)	14 (46.7)	-	-	-	-	30 (100)	0 (0)	0 (0)	0 (0)	18 (60)	12 (40)
** Galli 2021**	Stapler closure	46	67.99	44 (95.7)	7 (15.2)	-	-	23 (50)	-	40 (87)	-	-	-	-	-
	Manual closure	68	66.13	60 (88.2	11 (16.2)	-	-	19 (28)	-	48 (70.6)	-	-	-	-	-
** Sansa-Perna 2020**	Stapler closure	46	-	41 (89.13)	10 (21.74)	-	-	26 (56.52)	-	43 (93.48)	2 (4.35)	0 (0)	14 (30.43)	10 (21.74)	20 (43.48)
	Manual closure	80	-	74 (92.5)	21 (26.25)	-	-	39 (48.75)	-	70 (87.5)	1 (1.25)	0 (0)	8 (10)	24 (30)	47 (58.75)
** Öztürk 2019**	Stapler closure	21	60.05 (9.78)	20 (95.2)	-	-	-	0 (0)	-	21 (100)	-	-	-	-	-
	Manual closure	20	61.15 (7.6)	18 (90)	-	-	-	0 (0)	-	20 (100)	-	-	-	-	-
** Galletti 2018**	Stapler closure	16	42–92	36 (83.72)	-	-	-	-	-	15 (93.8)	0 (0)	0 (0)	0 (0)	25 (58.1)	18 (41.9)
	Manual closure	27								24 (88.9)					
** Ismi 2017**	Stapler closure	30	60.2 (7.2)	29 (96.67)	-	-	-	6 (20)	2 (6.67)	-	0 (0)	0 (0)	0 (0)	15 (50)	15 (50)
	Manual closure	40	60.05 (11.7)	38 (95)	-	-	-	7 (17.5)	4 (10)	-	0 (0)	0 (0)	0 (0)	15 (37.5)	25 (62.5)
** Dedivitis 2014**	Stapler closure	20	62 (11.1)	19 (95)	1 (5)	-	19 (95)	15 (75)	13 (65)	17 (85)	0 (0)	0 (0)	4 (20)	12 (60)	4 (20)
	Manual closure	67	62 (11.2)	61 (86.49)	3 (4.48)	-	65 (97.01)	15 (22.39)	17 (25.37)	64 (95.52)	0 (0)	0 (0)	3 (4.48)	46 (68.66)	18 (90)
** Miles 2013**	Stapler closure	16	60.3 (13.39)	38 (90.5)	5 (19.2)	25 (62.5)	38 (90.5)	9 (21.42)	-	-	-	-	-	-	-
	Manual closure	26													
**Sannikorn 2013**	Stapler closure	26	63.86 (10.21)	25 (96.1)	-	21 (80.8)	24 (92.3)	-	-	-	-	-	-	-	-
	Manual closure	26	62.04 (9.19)	24 (92.3)	-	24 (92.3)	25 (96.2)	-	-	-	-	-	-	-	-
** Calli 2011**	Stapler closure	61	59.7 (9.1)	60 (98.36)	-	-	-	0 (0)	-	-	-	-	-	-	-
	Manual closure	121	62.6 (8.2)	116 (95.87)	-	-	-	0 (0)	-	-	-	-	-	-	-
** Gonçalves 2009**	Stapler closure	30	58	28 (93.3)	-	-	-	14 (46.7)	10 (33.3)	54 (90)	-	-	-	-	-
	Manual closure	30	58.5	26 (86.7)	-	-	-	4 (13.3)	5 (16.7)		-	-	-	-	-

number (N), standard deviation (SD), years (Y)

### Risk of bias assessment

**Fig. 2:**
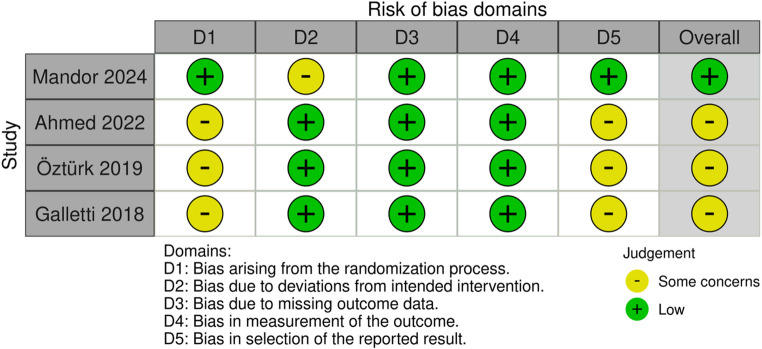
Risk of bias summary for randomized controlled trials (RCTs) using the Revised Cochrane Risk-of-Bias 2 (RoB-2) tool

The methodological quality and risk of bias were assessed using design-specific tools. For the included RCTs, the RoB-2 tool was utilized. One study was judged to have an overall low risk of bias. The remaining three RCTs were judged to have some concerns. These concerns were primarily related to potential biases arising from the randomization process and the selection of reported results (Fig. 2).

The quality of the 16 observational cohort studies was evaluated using the NOS. The overall quality was high, with 14 studies rated as Good and two as Fair (Supplementary Table 2).

### Primary outcome

**Fig. 3:**
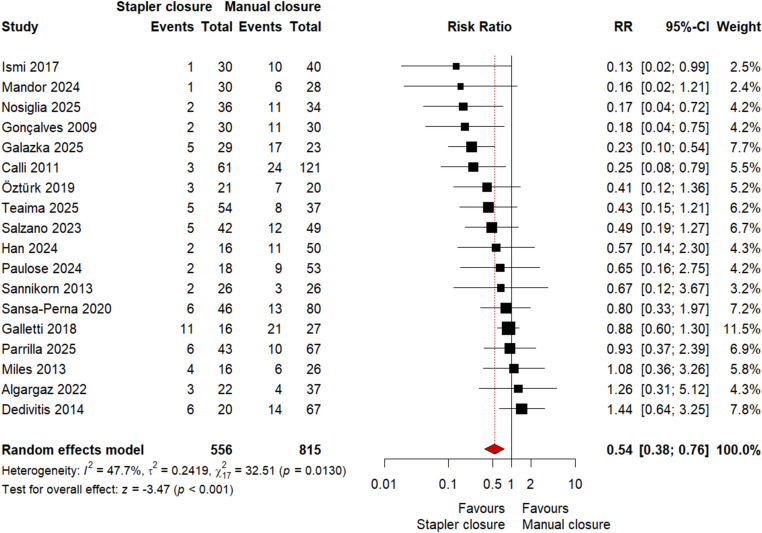
Forest plot comparing the incidence of pharyngocutaneous fistula (PCF) between stapler and manual closure groups

Our pooled analysis of PCF demonstrated a statistically significant reduction in risk for patients undergoing stapler closure compared to manual closure (RR = 0.54, 95% CI [0.38, 0.76], *p* < 0.001) with moderate heterogeneity (I² = 47.7%, *p* = 0.0130) **(**Fig. 3**)**.

**Fig. 4:**
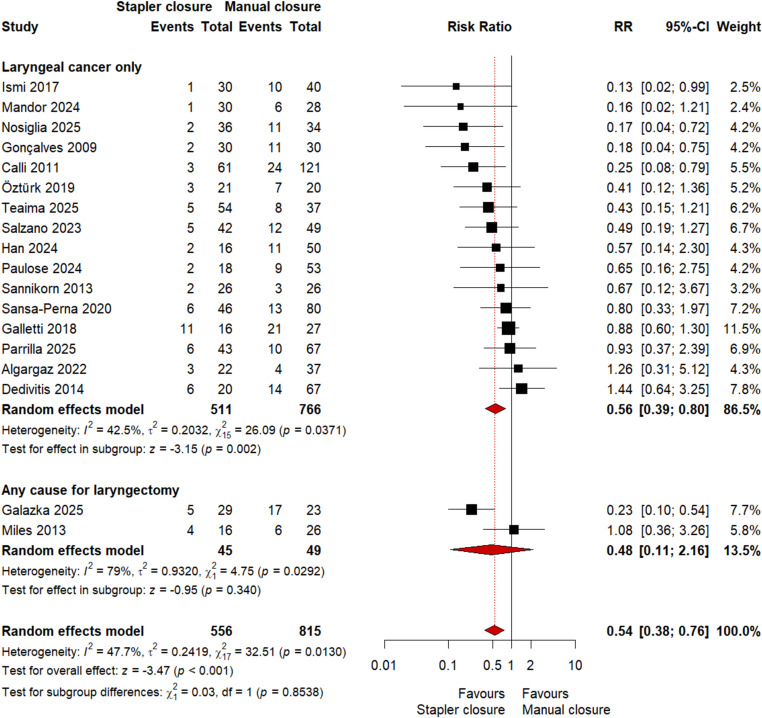
Subgroup analysis of pharyngocutaneous fistula (PCF) incidence based on cause of surgery (laryngeal cancer vs. other causes)

**Fig. 5:**
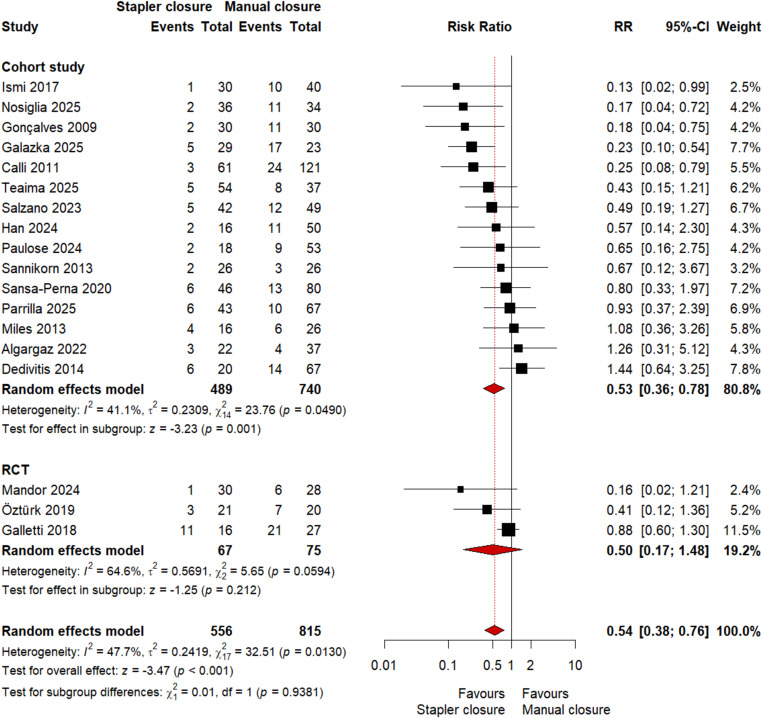
Subgroup analysis of pharyngocutaneous fistula (PCF) incidence based on study design (RCT vs. Cohort)

To investigate potential sources of heterogeneity, several subgroup analyses were performed. When stratified by the cause of surgery, a significant benefit for stapler closure was observed in patients with laryngeal cancer only (RR = 0.56, 95% CI [0.39, 0.80], *p* = 0.002; I² = 42.5%, *p* = 0.037), while the effect was not statistically significant for other causes (RR = 0.48, 95% CI [0.11, 2.16], *p* = 0.340; I² = 79%, *p* = 0.029) **(**Fig. 4**)**. Similarly, analysis by study design showed a significant risk reduction in cohort studies (RR = 0.53, 95% CI [0.36, 0.78], *p* = 0.001; I² = 41.1%, *p* = 0.049), but the pooled analysis from RCTs did not reach statistical significance (RR = 0.50, 95% CI [0.17, 1.48], *p* = 0.212; I² = 64.6%, *p* = 0.059) **(**Fig. 5**)**.

**Fig. 6:**
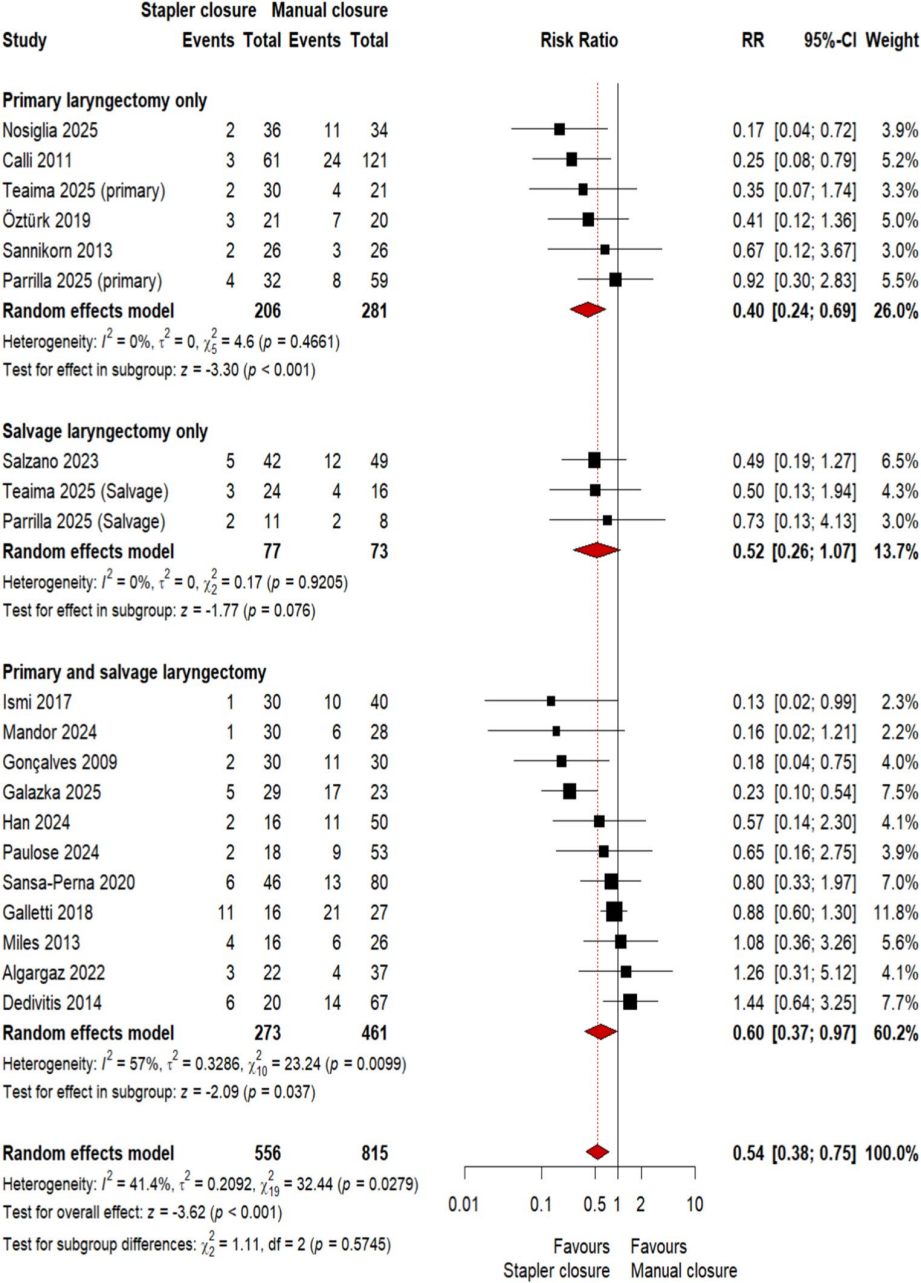
Subgroup analysis of pharyngocutaneous fistula (PCF) incidence based on laryngectomy type (Primary, Salvage, or Mixed)

Further stratification by laryngectomy type was performed to assess the effect in different clinical settings. The analysis revealed a significant benefit for stapler closure in cohorts of patients undergoing primary laryngectomy (RR = 0.40, 95% CI [0.24, 0.69], *p* > 0.001; I² = 0%, *p* = 0.466) and in mixed cohorts including both primary and salvage procedures (RR = 0.60, 95% CI [0.37, 0.97], *p* = 0.0037; I² = 57%, *p* = 0.009). However, in the subgroup of patients undergoing salvage laryngectomy only, the effect was not statistically significant (RR = 0.52, 95% CI [0.26, 1.07], *p* = 0.076; I² = 0%, *p* = 0.9205) **(**Fig. 6**)**.

**Fig. 7:**
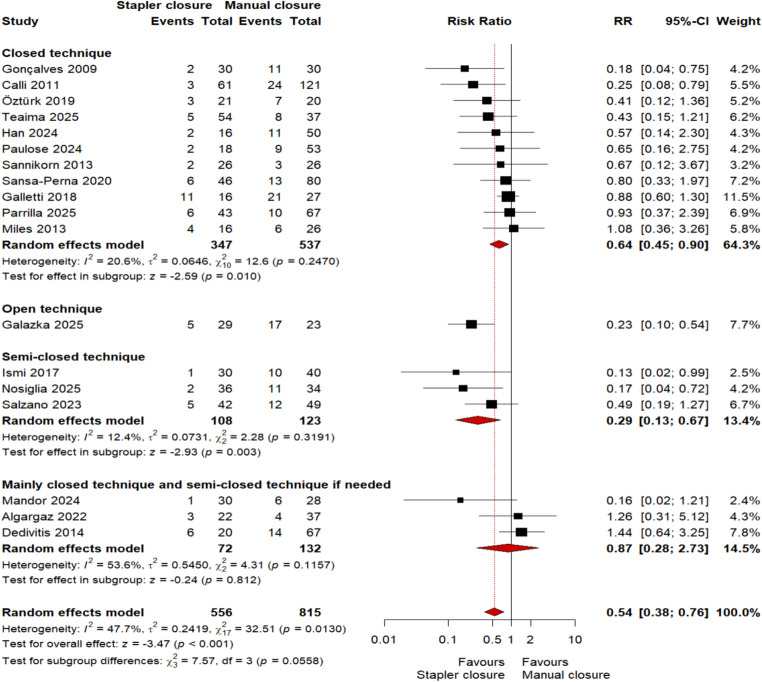
Subgroup analysis of pharyngocutaneous fistula (PCF) incidence based on stapler application technique

A subsequent subgroup analysis was conducted based on the specific stapler application technique used. A significant reduction in PCF risk was observed when a closed technique was used (RR = 0.64, 95% CI [0.45, 0.90], *p* = 0.010; I² = 20.6%, *p* = 0.2470) and with semi-closed techniques (RR = 0.29, 95% CI [0.13, 0.67], *p* = 0.003; I² = 12.4%, *p* = 0.3191). In contrast, the subgroup of “Mainly closed technique and semi-closed technique if needed” did not demonstrate a statistically significant effect on PCF risk (RR = 0.87, 95% CI [0.28, 2.73], *p* = 0.812; I² = 53.6%, *p* = 0.115). For the open technique subgroup, a significant risk reduction was also noted (RR = 0.23, 95% CI [0.10, 0.54]); however, this is based on data from a single included study **(**Fig. 7**)**.

An assessment for publication bias revealed some asymmetry in the funnel plot, and Egger’s test confirmed the presence of significant publication bias (*p* = 0.0244) **(**Supplementary Fig. 1**)**. Despite this, the robustness of the primary outcome was confirmed through a leave-one-out sensitivity analysis **(**Supplementary Fig. 2**)**.

### Secondary outcomes

#### Length of hospital stay

**Fig. 8:**
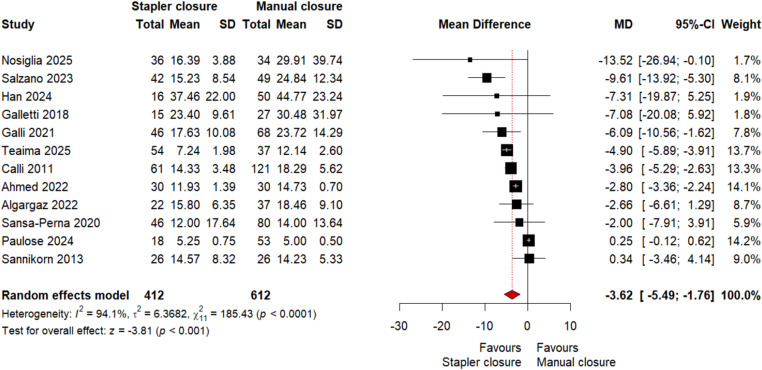
Forest plot comparing the length of hospital stay between stapler and manual closure groups

The analysis of the length of hospital stay revealed that patients in the stapler closure group had a significantly shorter hospitalization period compared to those undergoing manual closure (MD = −3.62, 95% CI [−5.49, −1.76], *p* < 0.001). This result was associated with substantial heterogeneity across the included studies (I² = 94.1%, *p* < 0.0001) **(**Fig. 8**)**.

To investigate this high heterogeneity, a subgroup analysis by laryngectomy type was performed. A significant reduction in hospital stay was observed in the primary laryngectomy subgroup (MD = −4.60, 95% CI [−5.42, −3.77], *p* > 0.001; I² = 2.4%, *p* = 0.380) and the salvage laryngectomy subgroup (MD = −7.90, 95% CI [−11.35, −4.45], *p* > 0.001; I² = 18.8%, *p* = 0.267). However, the effect was not statistically significant in the mixed cohort of primary and salvage laryngectomy (MD = −1.46, 95% CI [−3.64, 0.72], *p* = 0.190; I² = 93.9%, *p* = 0.001) **(**Supplementary Fig. 3).

A further subgroup analysis was conducted based on the stapler closure technique. A significant reduction in hospital stay was found in all subgroups: semi-closed technique (MD = −9.98, 95% CI [−14.08, −5.87], *p* > 0.001;; I² = 0%, *p* = 0.586), closed technique (MD = −2.58, 95% CI [−4.69, −0.48], *p* = 0.016; I² = 95.7%, *p* = 0.0001), and “mainly closed and semi-closed” (MD = −4.21, 95% CI [−7.55, −0.86], *p* = 0.014; I² = 21.1%, *p* = 0.260) (Supplementary Fig. 4). Despite these sources of variance, the leave-one-out sensitivity analysis confirmed the overall stability and robustness of the finding (Supplementary Fig. 5).

#### Operative time

The pooled analysis demonstrated a substantial and statistically significant reduction in operative time in the stapler closure group (MD = −52.60, 95% CI [−74.33, −30.86], *p* < 0.001). A high degree of heterogeneity was also present for this outcome (I² = 93.0%, *p* < 0.0001) (Supplementary Fig. 6).

Subgroup analysis by laryngectomy type showed a significant reduction in operative time across all three subgroups: primary laryngectomy (MD = −63.78, 95% CI [−100.84, −26.73], *p* < 0.001), primary and salvage laryngectomy (MD = −49.29, 95% CI [−74.06, −24.52], *p* < 0.001), and salvage laryngectomy (MD = −33.85, 95% CI [−60.76, −6.94], *p* = 0.014) (Supplementary Fig. 7).

Similarly, subgroup analysis by stapler closure technique showed a significant reduction in operative time for the semi-closed (MD = −39.94, 95% CI [−51.74, −28.13], *p* > 0.001) and closed (MD = −56.20, 95% CI [−88.58, −23.83], *p* > 0.001) subgroups. The effect was not significant for the “mainly closed and semi-closed” subgroup (MD = −61.05, 95% CI [−129.40, 7.30], *p* = 0.080) (Supplementary Fig. 8). The robustness of the overall outcome was confirmed by the leave-one-out analysis, showing that the result was not dependent on any individual study (Supplementary Fig. 9).

#### Time to start oral feeding

The pooled analysis of the time to initiate oral feeding showed that patients in the stapler closure group began eating significantly earlier than those in the manual closure group, with a mean difference of −3.35 days (CI [−6.06, −0.63], *p* = 0.016; I² = 81.3%, *p* = 0.0003) (Supplementary Fig. 10). The leave-one-out sensitivity analysis revealed that this finding lacked robustness. Specifically, the removal of the study by Teaima et al. (2025) rendered the pooled result non-significant (MD = −3.12, 95% CI [−6.50, 0.27], *p* = 0.0714) (Supplementary Fig. 11).

#### Surgical wound infection

The pooled analysis of four studies examining the incidence of surgical wound infection did not find a statistically significant difference between the stapler closure and manual closure groups (RR = 0.47, 95% CI [0.10, 2.14], *p* = 0.330; I² = 71.0%, *p* = 0.0160) (Supplementary Fig. 12).

A leave-one-out sensitivity analysis revealed that the overall result was not robust and was heavily influenced by a single study. Upon the removal of the study by Miles et al. (2013), the pooled risk ratio became statistically significant, strongly favoring the stapler closure group (RR = 0.22, 95% CI [0.09, 0.59], *p* = 0.0023) (Supplementary Fig. 13).

## Discussion

### Summary of key findings

This systematic review and meta-analysis synthesized current evidence to compare the efficacy and safety of stapler-assisted versus manual pharyngeal closure following total laryngectomy. The pooled results from 20 studies, including 1,545 patients, demonstrate that stapler-assisted closure offers significant clinical advantages over traditional manual suturing. The use of a mechanical stapler was associated with a statistically significant reduction in the incidence of PCF, as well as substantial decreases in both total operative time and the length of postoperative hospital stay.

However, our detailed subgroup analyses revealed important nuances. The protective effect against fistula formation was most evident in observational studies, primary laryngectomy settings, and when closed or semi-closed stapler techniques were employed. Notably, this benefit did not reach statistical significance in RCTs or in the subgroup of patients undergoing salvage laryngectomy alone. The reduction in operative time was a robust finding across nearly all clinical contexts, while the benefit of a shorter hospital stay was most pronounced in primary and salvage laryngectomy subgroups but was less clear in mixed patient cohorts due to high heterogeneity. Conversely, our analysis did not demonstrate a statistically significant benefit for stapler closure in reducing the time to initiation of oral feeding or the incidence of surgical wound infection. Importantly, both outcomes also lacked robustness, as their significance was highly dependent on individual studies.

### Interpretations of main findings

The results of this meta-analysis support and significantly update the conclusions drawn in previous systematic reviews by Lee et al. [8] and Chiesa-Estomba et al. [9], both of which identified stapler-assisted closure as a superior technique for reducing the incidence of PCF and shortening operative time. The biological plausibility for the reduced PCF rate observed in the stapler group is multifactorial. Unlike manual suturing, which can compromise mucosal microcirculation through irregular tension, tissue strangulation caused by suture knots, or excessive handling of the pharyngeal edges, the linear stapler applies a uniform double-staggered row of titanium staples [19, 26, 30, 31]. This mechanical precision facilitates a tension-free, hermetic seal that preserves neopharyngeal blood supply, a critical determinant in preventing local ischemic necrosis and subsequent fistulization [10, 32–34]. Furthermore, the utilization of closed or semi-closed techniques effectively isolates the pharyngeal lumen during resection, thereby preventing the spillover of saliva and pharyngeal secretions into the surgical field [3, 19, 35]. This containment significantly mitigates the risk of bacterial field contamination, which is a well-established precursor to deep neck space infection and fistula formation [18, 36]. Our subgroup analysis of stapler techniques provides a deeper insight missing from earlier meta-analyses. The significant reduction in PCF risk associated with closed and semi-closed techniques supports the hypothesis that avoiding wide pharyngotomy is protective [37, 38]. The closed technique allows for resection without exposing the aerodigestive tract, maintaining sterility [39, 40]. However, as noted by Galletti et al. [17], the closed technique carries a risk of trapping the epiglottis or achieving inadequate margins; the semi-closed technique, which utilizes a small keyhole incision for epiglottic traction, appears to offer a balance between oncological safety and minimizing contamination, potentially explaining its strong protective effect in our analysis [10]. Conversely, the open stapler technique, while offering direct visualization, reintroduces the risk of salivary contamination similar to manual closure, which may explain why the magnitude of benefit varies across technique subgroups [41–43].

While Chiesa-Estomba et al. [9] suggested overall benefits, our stratified analysis reveals a crucial divergence in the salvage laryngectomy setting. In patients undergoing salvage surgery exclusively, the reduction in PCF risk did not reach statistical significance. This finding aligns with the physiological challenges described by Salzano et al. [27] and Parrilla et al. [25]. In previously irradiated fields, the presence of chronic fibrosis, edema, and hypovascularity results in tissues that are less pliable and more prone to crumbling under compression [18, 44, 45]. These tissue characteristics may exceed the optimal compression range of standard stapler cartridges or prevent the staples from forming the ideal “B-shape” required for vascular preservation, thereby negating the mechanical advantages of the device [18, 27, 46].

The significant reduction in operative time is consistent with findings by Calli et al. [4] and Teaima et al. [28]. This efficiency is attributable to the stapler’s simultaneous cutting and sealing mechanism, which obviates the need for time-consuming, multi-layer manual suturing and hemostasis of the pharyngeal raw edges [37, 47]. This reduction in surgical duration, combined with the lower complication rate, directly translates into the significantly shorter length of hospital stay observed, a finding supported by the retrospective cohorts of Nosiglia et al. [23]. However, the moderate-to-high heterogeneity reported across these outcomes must be acknowledged. This variance is likely driven by differences in institutional discharge protocols, varying definitions of “operative time” (total surgery vs. pharyngeal closure time), and disparities in baseline patient characteristics (e.g., tumor stage and prior irradiation) across the included studies [20, 24].

Regarding the time to start oral feeding, while the pooled analysis suggested an earlier initiation in the stapler group, the leave-one-out sensitivity analysis revealed a lack of robustness; the significance was lost upon the removal of the study by Teaima et al. [28]. This instability suggests that the decision to initiate feeding is often protocol-driven rather than solely dependent on the immediate integrity of the closure. As noted by Sannikorn et al. [6], many surgeons adhere to rigid postoperative NPO (nothing by mouth) protocols regardless of the closure method, masking potential physiological benefits of the stapler.

Similarly, the analysis of surgical wound infection initially showed no significant difference. However, sensitivity analysis indicated that the result was heavily influenced by the study of Miles et al. [22], which reported a higher infection rate in the stapler group. When this study was removed, the pooled result shifted to favor the stapler significantly. This discrepancy may be due to the learning curve associated with stapler use in earlier cohorts or specific patient selection biases in that study. In contrast, more recent studies like Ismi et al. [3] demonstrate that the reduced operative time and minimized field contamination associated with staplers generally protect against surgical site infections.

Finally, The presence of publication bias, indicated by the significant Egger’s test and funnel plot asymmetry, suggests that studies with negative or non-significant findings regarding stapler use may be underreported. This is a common limitation in surgical literature where novel techniques are often published only when successful. Consequently, while the leave-one-out analyses confirm the consistency of the primary PCF reduction outcome, the magnitude of the benefit should be interpreted with caution, particularly in complex salvage scenarios where the evidence remains equivocal [15].

### Strengths and limitations

The primary strength of this systematic review and meta-analysis lies in its comprehensive scope and the inclusion of the most recent high-quality evidence. By incorporating 20 studies with a total sample size of 1,545 patients, including data from 2024 to 2025 publications, our analysis offers the most up-to-date evaluation of stapler-assisted pharyngeal closure. This expanded dataset allowed for robust subgroup analyses that were not possible in prior studies. Specifically, we were able to stratify outcomes by stapler technique (closed, semi-closed, open) and surgical indication (primary vs. salvage laryngectomy), providing detailed insights that address previous gaps in the literature. Furthermore, the application of rigorous leave-one-out sensitivity analyses for all outcomes enhances the reliability of our findings, distinguishing robust clinical benefits from those driven by single-study outliers.

However, limitations must be acknowledged. First, the substantial heterogeneity observed in secondary outcomes, particularly operative time and length of hospital stay. This heterogeneity likely stems from differences in discharge protocols, definitions of surgical time (total vs. closure-specific), and baseline patient characteristics, as noted in the diverse populations of Öztürk et al. [24] and Teaima et al. [28]. Second, the majority of included studies were observational cohorts rather than RCTs. While we used the NOS to ensure high quality among observational studies, the lack of randomization introduces inherent selection bias. Surgeons may have preferentially selected stapler closure for easier cases with less tumor extension or better tissue quality, potentially inflating the success rates of the mechanical technique [18, 21]. Additionally, several confounding variables that significantly influence healing were not consistently reported across the included studies, preventing quantitative analysis. These include the surgeon’s experience level (volume of cases), specific perioperative antibiotic prophylaxis protocols, and the method of postoperative feeding (nasogastric tube vs. pharyngostomy). Furthermore, in salvage cases, the time interval between the completion of radiation/chemoradiation and the salvage surgery was rarely specified, despite its known impact on tissue viability. Finally, the presence of publication bias, confirmed by Egger’s test for the primary outcome, indicates that studies with negative or non-significant findings regarding stapler use may be underrepresented in the literature, a common issue in surgical innovation research [15].

### Clinical implications and advances in knowledge

The findings of this study have direct implications for surgical decision-making in head and neck oncology. The demonstration of a significant reduction in PCF rates with stapler-assisted closure supports its adoption as a standard of care for primary total laryngectomy. Surgeons can confidently employ closed or semi-closed stapler techniques to minimize field contamination and optimize mucosal healing. The substantial reduction in operative time—averaging nearly one hour—combined with shorter hospital stays presents a clear argument for the cost-effectiveness of staplers, potentially offsetting the higher device costs through reduced operating room utilization and bed occupancy.

Critically, this review advances current knowledge by delineating the limitations of stapler use in salvage laryngectomy. Contrary to the broad endorsements of earlier reviews, our stratified analysis indicates that the protective effect against PCF is not statistically significant in salvage settings alone. This suggests that in patients with prior irradiation or chemoradiation, where tissue fibrosis and hypovascularity compromise staple integrity, the choice of closure technique should be individualized. In these high-risk scenarios, surgeons should not rely solely on the stapler to prevent fistulas but should consider it primarily for its time-saving benefits, while remaining vigilant for potential complications. Furthermore, our results caution against modifying postoperative care pathways, such as early oral feeding, based solely on the use of a stapler. The lack of robust evidence supporting earlier feeding in the stapler group implies that oral intake initiation should continue to be guided by clinical assessment and institutional protocols rather than the closure method itself. Finally, while staplers provide a standardized closure, their use should not preclude intraoperative verification of the repair’s integrity. As noted by Tyson et al., [48] checking for ‘water-tightness’ remains a vital step often overlooked in modern protocols. Techniques such as the Hydrogen Peroxide Test or the instillation of sterile saline combined with AMBU (Artificial Manual Breathing Bag) insufflation can identify immediate occult leaks, allowing for immediate reinforcement of the staple line before wound closure.

Future research must pivot toward high-quality, multicenter RCTs specifically designed to address the complexities of salvage laryngectomy. Given that our analysis found no statistical benefit for PCF reduction in salvage cases, future studies should be stratified by the type and dosage of prior treatment (e.g., chemoradiation vs. radiation alone) to determine if specific stapler cartridge heights or tissue-reinforcement materials can overcome radiation-induced fibrosis and hypovascularity. Additionally, there is a critical need for comprehensive cost-utility analyses to definitively establish whether the higher upfront cost of stapling devices is economically neutralized by the significant reductions in operative time and hospital bed occupancy, a variable that remains inconsistent across healthcare systems. Furthermore, long-term functional outcomes require more rigorous assessment; specifically, future investigations should evaluate the impact of the rigid staple line on neopharyngeal motility, stricture formation, and the long-term viability of primary tracheoesophageal puncture for voice restoration, as current data focuses predominantly on immediate postoperative complications rather than chronic functional rehabilitation. Moreover, future large-scale RCTs should consider the potential impact of biological sex on pharyngeal healing and the incidence of postoperative complications, particularly PCF. Sex-related differences have been shown to influence risk events in other populations [49, 50]. Finally, comparing the closed versus semi-closed techniques in a controlled setting would help standardize surgical protocols and minimize the technical heterogeneity that currently confounds the assessment of field contamination risks.

### Conclusion

Stapler-assisted pharyngeal closure represents a significant advancement in the surgical management of laryngeal cancer. This meta-analysis confirms that, compared to manual suturing, the use of a linear stapler significantly reduces the incidence of PCF, total operative time, and length of hospital stay in the overall population. The closed and semi-closed techniques appear particularly effective in minimizing infectious complications by preventing salivary contamination. However, the clinical benefit is most robust in patients undergoing primary laryngectomy. In the setting of salvage laryngectomy, while the stapler offers efficiency advantages, it is not significantly superior to manual closure in preventing fistula formation, likely due to radiation-induced tissue changes. Consequently, stapler-assisted closure should be the preferred technique for suitable candidates, particularly in primary cases, while its application in salvage surgery requires careful patient selection and surgical judgment. Future research should focus on large-scale RCTs specifically stratified by prior treatment history to further refine indications for the salvage population.

## Electronic Supplementary Material

Below is the link to the electronic supplementary material.

Supplementary Material 1 (DOCX 4.28 MB)

## Data Availability

The datasets supporting the conclusions of this article are included within the article and its additional file.
